# Percutaneous compression screw fixation for hinge fractures in distal femoral osteotomy does not compromise radiographic or clinical outcomes: A retrospective case‐control study

**DOI:** 10.1002/jeo2.70830

**Published:** 2026-06-30

**Authors:** Victor Relot, Ernest Siret, Thibault Liguori, Romir Patel, Antoine Piercecchi, Matthieu Ollivier

**Affiliations:** ^1^ Assistance publique des Hôpitaux de Marseille, APHM, Orthopedic Surgery Department, APHM, CNRS, ISM, Institute of Movement Sciences Aix Marseille University Marseille France

**Keywords:** complications, distal femoral osteotomy, hinge fracture, knee osteotomy, screw fixation

## Abstract

**Purpose:**

The purpose of this study was to compare radiographic and clinical outcomes between patients undergoing distal femoral osteotomy (DFO) with intraoperative hinge fracture treated with percutaneous compression screw fixation and those without hinge fracture.

**Methods:**

This retrospective case‐control study included patients undergoing DFO for coronal plane deformity correction between January 2019 and May 2023, with minimum 2‐year follow‐up. Patients sustaining a hinge fracture treated with percutaneous compression screw fixation constituted the case group; patients without hinge fracture served as unmatched controls. Radiographic parameters—including coronal correction (ΔHKA: change in hip–knee–ankle angle), alignment maintenance and radiographic union—and patient‐reported outcome measures (Knee Injury and Osteoarthritis Outcome Score, Lysholm score, Simple Knee Value, UCLA Activity Score, NRS pain score) were assessed pre and postoperatively. Statistical significance was set at *p* < 0.05.

**Results:**

Of 130 patients screened, 67 were included: 21 in the hinge fracture group and 46 controls. Mean age was 41.8 ± 13.9 years, 75% were male and mean BMI was 26.8 ± 4.5 kg/m^2^. Baseline demographics were comparable except for osteoarthritis grade, which was more advanced in the hinge fracture group (*p* = 0.015). Mean follow‐up was 3.5 ± 1.1 years. Coronal correction did not differ between groups (Δhip–knee–ankle angle: 7.2 ± 3.1° vs. 8.1 ± 3.8°; *p* = 0.486), and alignment maintenance was comparable. All osteotomies achieved radiographic union. Patient reported outcome measures improved significantly in both groups with no between‐group differences at final follow‐up (Lysholm: 78.3 ± 21.6 vs. 72.7 ± 26.2; *p* = 0.481). Progressive weight‐bearing initiated at 3 weeks was not associated with loss of correction or delayed union.

**Conclusion:**

DFO‐associated hinge fractures treated with percutaneous compression screw fixation were not associated with inferior radiographic or clinical outcomes compared with patients without hinge fracture. Progressive weight‐bearing from 3 weeks was safe, supporting this technique as a reliable intraoperative management strategy.

**Level of Evidence:**

Level III, retrospective comparative cohort.

**Abbreviations**
DFO: distal femoral osteotomy
HKA: hip–knee–ankle angle
JLCA: joint line convergence angle
JLO: joint line obliquity
KOOS: Knee Injury and Osteoarthritis Outcome Score
LDFA: lateral distal femoral angle
MPTA: medial proximal tibial angle
NRS: numerical rating scale
SKV: simple knee value
UCLA: University of California Los Angeles

## INTRODUCTION

Distal femoral osteotomy (DFO) is a well‐established joint‐preserving procedure for the management of coronal plane malalignment in young and active patients, reducing pain, improving functional outcomes and delaying the need for total knee arthroplasty [18, 22, 23]. Both medial closing‐wedge and lateral opening‐wedge techniques have demonstrated favourable long‐term survival when accurate correction and stable fixation are achieved [6, 14, 34].

One of the most frequently reported intraoperative complications is hinge fracture, occurring at the cortical pivot point during opening‐wedge or closing‐wedge osteotomy. Hinge fracture incidence ranges from 10% to 57%, depending on surgical technique, hinge location, correction magnitude and definition criteria [29, 31, 32]. Medial hinge disruption may compromise construct stability, potentially leading to loss of correction, rotational instability, delayed union or nonunion [12, 20, 21, 27]. These concerns have led some authors to recommend prolonged protected weight‐bearing or revision fixation in cases of hinge fracture [16, 17].

However, emerging evidence suggests that hinge fracture does not inevitably lead to inferior outcomes when appropriate stabilisation is performed [11]. Supplemental compression screw fixation has been proposed to restore hinge continuity and improve mechanical stability, with biomechanical studies demonstrating improved axial and rotational stiffness [7, 8]. In contrast to high tibial osteotomy, where hinge fractures have been extensively investigated, clinical evidence evaluating their management in DFO remains limited and optimal rehabilitation protocols remain incompletely defined.

The primary aim of this study was to compare radiographic outcomes—including coronal correction and alignment maintenance—between DFO patients with intraoperative hinge fracture treated with percutaneous compression screw fixation and those without hinge fracture. The primary hypothesis was that hinge fracture stabilisation would not compromise correction maintenance or bone healing. The secondary aim was to compare clinical outcomes and functional recovery between groups; the secondary hypothesis was that patient‐reported outcomes would be comparable between patients with stabilised hinge fractures and those without.

## METHODS

This retrospective single‐centre case–control study was conducted at a tertiary referral institution specialising in knee preservation surgery. Institutional review board approval was obtained before study initiation (reference number: CSE_PADS23_021_DGR), and the study was performed in accordance with the Declaration of Helsinki [33]. All patients undergoing DFO between January 2019 and May 2023 were identified through the institutional surgical database. Clinical records, operative reports and radiographic data were reviewed retrospectively. Patients were eligible for inclusion if they were aged 16 years or older at the time of surgery, underwent DFO for coronal plane malalignment, had no significant ligamentous instability requiring concomitant reconstruction and had complete clinical and radiographic follow‐up for a minimum of 24 months. Radiographic quality was assessed before inclusion; patients with inadequate full‐length standing radiographs precluding reliable measurement were excluded. Patients were excluded if they had a history of prior infection involving the distal femur or knee, underwent rotational osteotomy without coronal correction, had prior surgery to the ipsilateral knee or underwent revision osteotomy procedures. Osteoarthritis severity was graded according to the Kellgren–Lawrence classification on preoperative weight‐bearing radiographs, using a four‐grade scale (grade 0: no radiographic features of osteoarthritis; grade 1: doubtful joint‐space narrowing with possible osteophytic lipping; grade 2: definite osteophytes and possible narrowing of joint space; grade 3: moderate multiple osteophytes, definite narrowing of joint space and some sclerosis). Grading was performed by the same fellowship‐trained orthopaedic surgeon who conducted all radiographic measurements, blinded to clinical outcomes [13].

Patients were divided into two groups based on intraoperative hinge integrity. The hinge fracture group consisted of patients who sustained a hinge fracture during osteotomy and were treated with percutaneous compression screw fixation, while the control group included patients without hinge fracture. Controls were not matched but were selected from the same consecutive surgical cohort. Hinge fracture was defined as any cortical disruption at the osteotomy hinge identified intraoperatively under fluoroscopic guidance. In this series, all hinge fractures were detected during the index procedure; none were identified solely on postoperative radiographs. All hinge fractures identified intraoperatively were stabilised immediately using percutaneous compression screw fixation.

All procedures were performed by two experienced fellowship‐trained knee surgeons using standardised surgical techniques. The choice between medial closing‐wedge and lateral opening‐wedge DFO was determined based on deformity characteristics and surgeon preference. In lateral opening‐wedge osteotomy, a lateral approach to the distal femur was used and the osteotomy was performed under fluoroscopic guidance while preserving the medial cortical hinge whenever possible. Gradual opening of the osteotomy was achieved using calibrated osteotomy wedges until the planned correction was obtained. In medial closing‐wedge osteotomy, two converging osteotomy cuts were performed from the medial aspect of the distal femur under fluoroscopic control, followed by removal of the bone wedge and controlled closure of the osteotomy. In all cases, a biplanar osteotomy was performed by adding an ascending anterior cut in the frontal plane, irrespective of the opening‐ or closing‐wedge configuration, to improve rotational stability and increase bone contact surface area at the osteotomy site. Definitive fixation was performed using anatomically contoured locking plates (ActivMotion plate, NewClip Technics) with locking screw fixation to ensure stable osteosynthesis.

Hinge fracture was defined as any cortical disruption at the osteotomy hinge identified intraoperatively under fluoroscopic guidance. In this series, all hinge fractures were detected during the index procedure; none were identified solely on postoperative radiographs.

**Figure 1:**
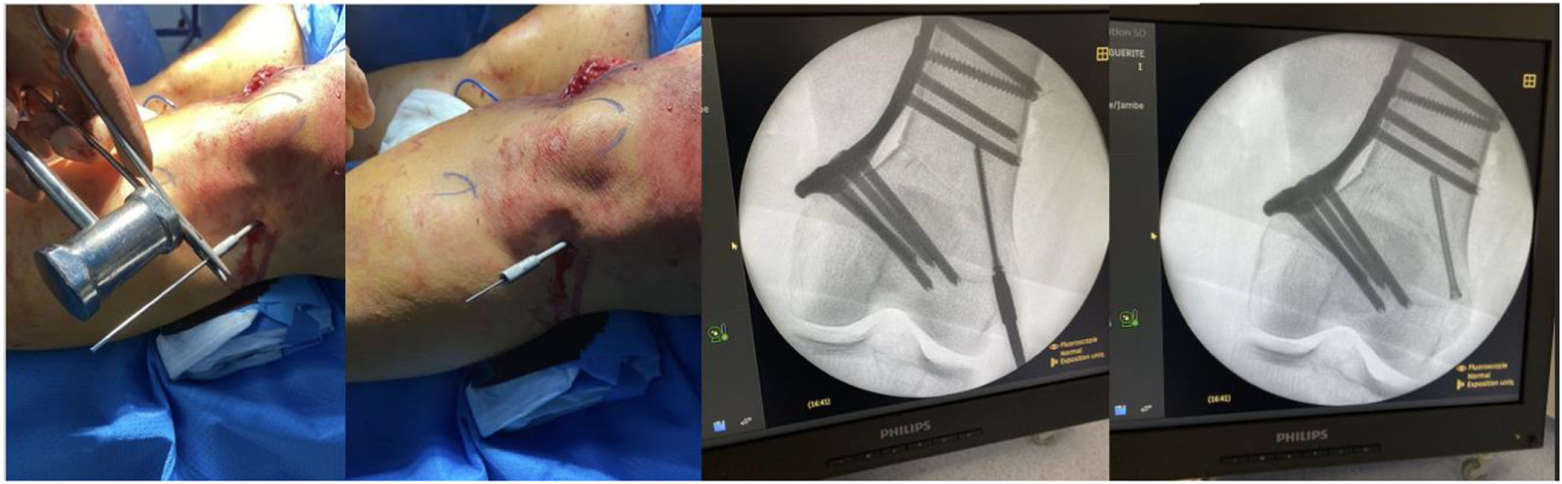
Intraoperative photograph and fluoroscopic images demonstrating percutaneous compression screw fixation of a hinge fracture during lateral closing‐wedge distal femoral osteotomy (DFO). The screw is inserted distally in an ascending trajectory, oriented tangentially to the fracture plane. All cases depicted are left knees. Intraoperative photographs have been reoriented to a standardised vertical position for clarity.

In cases of hinge fracture, supplemental fixation was performed using percutaneous headless compression screws (4.0 mm, NewClip Technics, France) detailed description of the surgical technique is provided in Appendix 1. Under fluoroscopic guidance, the compression screw was inserted across the hinge fracture site to restore cortical continuity and enhance mechanical stability before completion of definitive plate fixation (Figure 1). This supplemental fixation was intended to convert an unstable hinge fracture into a mechanically stable construct capable of supporting physiological loading conditions.

All patients followed a standardised postoperative rehabilitation protocol. Patients without hinge fracture were allowed immediate protected weight‐bearing—defined as partial weight‐bearing with crutches—with progressive advancement to full weight‐bearing at 6 weeks postoperatively. Patients with hinge fracture treated with compression screw fixation followed a more conservative protocol consisting of toe‐touch weight‐bearing during the first 3 weeks, followed by progressive partial weight‐bearing advancement to full weight‐bearing at 6 weeks. Passive and active range‐of‐motion exercises were initiated immediately after surgery in both groups.

Radiographic evaluation was performed using standardised full‐length standing anteroposterior radiographs obtained preoperatively, at the first postoperative outpatient visit (mean 3 months postoperatively, once full weight‐bearing had been achieved), and at final follow‐up. Standard non‐weight‐bearing anteroposterior and lateral knee radiographs were obtained before discharge to confirm implant positioning and assess hinge integrity; these images were not used for alignment measurement. Only full‐length standing anteroposterior radiographs of adequate technical quality—defined as complete visualisation of both hip and ankle joints with the patella facing forward—were retained for analysis; patients with inadequate imaging were excluded from the study. Radiographic parameters included hip–knee–ankle angle (HKA) [10], lateral distal femoral angle (LDFA), medial proximal tibial angle (MPTA), joint line obliquity (JLO) and joint line convergence angle (JLCA) [25]. All measurements were performed using calibrated digital radiographs and dedicated planning software (mediCAD, Hectec GmbH, Germany). Measurements were conducted by a fellowship‐trained orthopaedic surgeon who was blinded to clinical outcomes in order to minimise observer bias. Intraobserver reliability was assessed by performing all measurements twice at separate time points and calculating the intraclass correlation coefficient (ICC) using a two‐way mixed‐effects absolute agreement model. Interobserver reliability assessment was not performed. Intraobserver reliability for radiographic measurements was excellent, with an overall ICC of 0.91 (95% confidence interval [CI]: 0.86–0.95), indicating high measurement consistency. Radiographic union was defined as bridging callus formation visible on at least three of four cortices on anteroposterior and lateral radiographs, combined with absence of clinical symptoms during weight‐bearing, as previously described [1]. Loss of correction was defined as a change greater than 2° in HKA angle between immediate postoperative and final follow‐up radiographs, a threshold exceeding the reported measurement error of full‐length radiographic alignment assessment and considered clinically relevant for load redistribution across the knee joint in our practice.

Clinical outcomes were assessed using validated patient‐reported outcome measures, including the Knee Injury and Osteoarthritis Outcome Score (KOOS) [4, 26], Lysholm score [2, 30], simple knee value (SKV) [4, 24], University of California Los Angeles (UCLA) activity score [19] and numerical rating scale (NRS) for pain [5, 28]. The minimal clinically important difference (MCID) was defined as 10 points for KOOS subscales [9, 15], 10 points for the Lysholm score [26], 10 points for the SKV [24] and 2 points for the NRS pain score [5]. Patient satisfaction was also evaluated at final follow‐up.

Patients underwent standardised clinical and radiographic follow‐up at 6 weeks, 3 months, 6 months, 12 months and final follow‐up at a minimum of 24 months.

Postoperative rehabilitation protocols differed between groups with respect to timing of weight‐bearing.

Continuous variables were presented as mean and standard deviation, and normality was assessed using the Shapiro–Wilk test. Continuous variables were compared using Student's *t*‐test or Mann–Whitney *U*‐test, depending on data distribution, while categorical variables were compared using chi‐square test or Fisher's exact test. Statistical significance was set at *p* < 0.05. All statistical analyses were performed using EasyMedStat software (version 3.42, France).

A formal a priori power calculation was not performed, given the retrospective nature of the study. Based on a MCID of 2° in HKA angle, a standard deviation of 3°, an alpha of 0.05 and a beta of 0.20, a minimum of 18 patients per group would be required to detect this difference, suggesting adequate power for the primary radiographic outcome. However, the study may have been underpowered to detect small differences in patient‐reported outcome measures.

## RESULTS

A total of 130 patients who underwent DFO during the study period were screened for eligibility. Of the 63 excluded patients, 35 had insufficient follow‐up (< 24 months), 27 underwent rotational femoral osteotomy without coronal correction and 1 had a history of prior infection at the operative site. The remaining 67 patients were included in the final analysis. Among these, 21 patients (31%) sustained an intraoperative hinge fracture and were treated with percutaneous compression screw fixation, while 46 patients (69%) did not sustain a hinge fracture and served as the control group. The mean age of the overall cohort was 41.8 ± 13.9 years (range, 17–71 years), and 50 patients (75%) were male. The mean follow‐up duration was 3.5 ± 1.1 years (range, 2.0–5.8 years). Baseline demographic characteristics, including age, sex, body mass index and osteotomy type, were not significantly different between groups (all *p* > 0.050). Osteoarthritis severity, graded according to the Kellgren–Lawrence classification, differed between groups, with the hinge fracture group demonstrating a higher proportion of Kellgren–Lawrence grade ≥2 osteoarthritis [13] (*p* = 0.015) (Table 1). Concomitant procedures were performed in 26 patients (39%), including double‐level osteotomy (DFO + high tibial osteotomy [HTO]) in 18 patients (27%), arthroscopy in 5 patients (8%), ligament reconstruction in 5 patients (8%; 3 anterior cruciate ligament reconstructions, 2 posterolateral corner reconstructions) and meniscal procedures in 3 patients (5%; 2 partial meniscectomies, 1 meniscal repair). Distribution of concomitant procedures was not significantly different between groups (all *p* > 0.050).

**Table 1:** Clinical and demographic characteristics.

Characteristic	Hinge fracture group (*n* = 21)	Non fracture group (*n* = 46)	Overall (*n* = 67)	*p*‐Value
Demographics				
Age (years), mean ± SD	46.1 ± 11.8 [40.7–51.5]	39.9 ± 14.5 [35.6–44.2]	41.8 ± 13.9 [38.4–45.2]	0.089
Male sex, *n* (%)	15 (71.4) [52.1–85.8]	35 (76.1) [61.2–86.5]	50 (74.6) [62.9–83.6]	0.765
BMI (kg/m^2^), mean ± SD	27.4 ± 5.4 [25.0–29.9]	26.5 ± 4.1 [25.3–27.7]	26.8 ± 4.5 [25.7–27.9]	0.450
Osteoarthritis grade, *n* (%)				0.015*
Grade 0	3 (14.3) [5.0–34.6]	21 (45.6) [31.8–60.2]	24 (35.8) [25.2–48.1]	
Grade 1	8 (38.1) [21.2–58.3]	14 (30.4) [18.8–45.4]	22 (32.8) [22.6–45.0]	
Grade 2	9 (42.9) [24.5–63.5]	7 (15.2) [7.5–28.5]	16 (23.9) [15.2–35.4]	
Grade 3	1 (4.8) [0.8–22.7]	4 (8.7) [3.4–20.3]	5 (7.5) [[3.2–16.4]	
Osteotomy type, *n* (%)				
LCW‐DFO	14 (66.7) [46.0–82.8]	30 (65.2) [50.1–77.7]	44 (65.7) [53.7–75.9]	>0.999
LOW‐DFO	1 (4.8) [0.8–22.7]	4 (8.7) [3.4–20.3]	5 (7.5) [3.2–16.4]	0.735
MCW‐DFO	6 (28.6) [14.6–48.9]	12 (26.1) [15.6–40.6]	18 (26.9) [17.9–38.3]	0.553
Combined procedures, *n* (%)				
DFO + HTO	7 (33.3) [17.2–54.0]	11 (23.9) [13.7–38.6]	18 (26.9) [17.9–38.3]	0.553
Patient‐specific instrumentation	5 (23.8) [10.6–45.1]	16 (34.8) [22.6–49.2]	21 (31.3) [21.2–43.4]	0.641
Associated procedures, *n* (%)				
Arthroscopy	2 (9.5) [2.7–28.9]	3 (6.5) [2.3–17.6]	5 (7.5) [3.2–16.4]	0.089
Ligament reconstruction	0 (0.0)	5 (10.9) [4.8–23.1]	5 (7.5) [3.2–16.4]	0.173
Meniscal procedures	1 (4.8) [0.8–22.7]	2 (4.4) [1.2–14.8]	3 (4.5) [1.6–12.5]	>0.999

*Note*: Values are presented as mean ± standard deviation [95% confidence interval] or number (percentage) [95% confidence interval].

Abbreviations: BMI, body mass index; DFO, distal femoral osteotomy; HTO, high tibial osteotomy; LCW, lateral closing wedge; LOW, lateral opening wedge; MCW, medial closing wedge; SD, standard deviation.

* Significant at *p* < 0.05.

Radiographic alignment parameters were assessed preoperatively, postoperatively, and at final follow‐up (Tables 2 and 3). The mean change in ΔHKA was 7.2 ± 3.1° in the hinge fracture group and 8.1 ± 3.8° in the control group, with no statistically significant difference between groups (*p* = 0.486). Changes in ΔLDFA, ΔMPTA, ΔJLO and ΔJLCA were also measured, and no statistically significant differences were observed between groups for any of these parameters (all *p* > 0.05). Comparison between immediate postoperative and final follow‐up radiographs demonstrated maintenance of correction in both groups. Radiographic union was observed in all 67 patients (100%). No cases of delayed union, nonunion, hardware failure or revision surgery related to osteotomy healing were recorded in either group during the follow‐up period.

**Table 2:** Radiographic corrections in varus knees.

Parameter	Time point	Hinge fracture group (*n* = 14)	Non fracture group (*n* = 28)	*p*‐Value
HKA angle (°)	Preoperative	170.4 ± 3.4 [168.4–172.4]	170.1 ± 2.9 [169.0–171.2]	0.796
	Postoperative	177.5 ± 2.8 [175.9–179.1]	178.1 ± 2.8 [177.0–179.2]	0.467
MPTA (°)	Preoperative	85.1 ± 2.7 [83.5–86.7]	84.3 ± 2.2 [83.5–85.1]	0.318
	Postoperative	87.4 ± 2.4 [86.0–88.8]	87.6 ± 2.6 [86.6–88.6]	0.770
LDFA (°)	Preoperative	92.2 ± 3.2 [90.3–94.1]	91.9 ± 3.2 [90.7–93.1]	0.976
	Postoperative	87.7 ± 2.2 [86.4–89.0]	87.5 ± 2.9 [86.4–88.6]	0.833
JLO (°)	Preoperative	2.6 ± 1.8 [1.6–3.6]	2.0 ± 2.0 [1.3–2.7]	0.518
	Postoperative	2.0 ± 2.2 [0.7–3.3]	2.1 ± 3.0 [1.0–3.2]	0.889
JLCA (°)	Preoperative	1.5 ± 3.0 [−0.2 to 3.2]	2.1 ± 2.9 [1.0–3.2]	0.471
	Postoperative	2.4 ± 2.1 [1.2–3.6]	1.7 ± 1.8 [1.0–2.4]	0.255

*Note*: Data presented as mean ± SD [95% confidence interval].

Abbreviations: HKA, hip‐knee‐ankle angle; JLCA, joint line convergence angle; JLO, joint line obliquity; LDFA, lateral distal femoral angle; MPTA, medial proximal tibial angle; SD, standard deviation.

**Table 3:** Radiographic corrections in valgus knees.

Parameter	Time point	Hinge fracture group (*n* = 7)	Non fracture group (*n* = 18)	*p*‐Value
HKA angle (°)	Preoperative	188.5 ± 3.3 [185.5–191.5]	187.1 ± 3.7 [185.3–188.9]	0.386
	Postoperative	180.8 ± 3.2 [177.8–183.8]	179.5 ± 2.3 [178.4–180.6]	0.360
MPTA (°)	Preoperative	89.7 ± 2.7 [87.2–92.2]	91.3 ± 3.0 [89.9–92.7]	0.431
	Postoperative	88.7 ± 4.3 [84.7–92.7]	88.9 ± 2.6 [87.7–90.1]	0.904
LDFA (°)	Preoperative	82.0 ± 2.0 [80.1–83.9]	83.9 ± 2.7 [82.6–85.2]	0.116
	Postoperative	88.2 ± 3.0 [85.4–91.0]	89.1 ± 2.9 [87.7–90.5]	0.599
JLO (°)	Preoperative	4.2 ± 1.3 [3.0–5.4]	1.6 ± 3.3 [0.0–3.2]	0.056
	Postoperative	2.1 ± 4.9 [−2.4 to 6.6]	1.2 ± 2.7 [−0.1 to 2.5]	0.620
JLCA (°)	Preoperative	−0.9 ± 2.2 [−2.9 to 1.1]	0.7 ± 2.4 [−0.4 to 1.8]	0.134
	Postoperative	0.7 ± 1.6 [−0.8 to 2.2]	0.1 ± 1.7 [−0.7 to 0.9]	0.529

*Note*: Data presented as mean ± SD [95% confidence interval].

Abbreviations: HKA, hip‐knee‐ankle angle; JLCA, joint line convergence angle; JLO, joint line obliquity; LDFA, lateral distal femoral angle; MPTA, medial proximal tibial angle; SD, standard deviation.

Clinical outcomes were evaluated using validated patient‐reported outcome measures (Table 4). In the hinge fracture group, the mean KOOS pain score improved from 50.8 ± 24.5 preoperatively to 74.9 ± 23.6 at final follow‐up, and the mean Lysholm score improved from 45.3 ± 22.7 to 74.8 ± 24.2. The mean SKV improved from 33.6 ± 20.8 to 73.5 ± 20.7. Similar improvements were observed in the control group. At final follow‐up, no statistically significant differences were observed between groups for any KOOS subscale, Lysholm score, SKV, UCLA activity score or NRS pain score (all *p* > 0.05). Improvements in both groups exceeded the established MCID thresholds for all outcome measures, including 10 points for KOOS subscales and Lysholm score and 2 points for NRS pain score.

**Table 4:** Preoperative and postoperative clinical scores.

Score	Time point	Hinge fracture group (*n* = 21)	Non fracture group (*n* = 46)	Overall (*n* = 67)	*p*‐Value
KOOS—Symptoms and stiffness	Preoperative	49.4 ± 21.9 [40.0–58.8]	55.1 ± 23.3 [48.4–61.8]	53.5 ± 22.5 [48.1–58.9]	0.392
	Postoperative	68.6 ± 21.7 [59.3–77.9]	72.3 ± 22.0 [65.9–78.7]	70.9 ± 21.5 [65.8–76.0]	0.602
KOOS—Pain	Preoperative	53.5 ± 25.0 [42.8–64.2]	49.3 ± 25.0 [42.1–56.5]	50.8 ± 24.5 [45.0–56.6]	0.601
	Postoperative	76.7 ± 23.3 [66.7–86.7]	73.8 ± 24.6 [66.7–80.9]	74.9 ± 23.6 [69.3–80.5]	0.990
KOOS—Function (ADL)	Preoperative	59.5 ± 25.3 [48.7–70.3]	55.3 ± 24.1 [48.3–62.3]	56.7 ± 24.1 [51.0–62.4]	0.597
	Postoperative	81.1 ± 22.6 [71.4–90.8]	75.7 ± 23.9 [68.8–82.6]	77.7 ± 23.1 [72.2–83.2]	0.715
KOOS—Sports and recreation	Preoperative	28.3 ± 30.4 [15.3–41.3]	20.7 ± 21.2 [14.6–26.8]	23.4 ± 24.4 [17.6–29.2]	0.563
	Postoperative	51.7 ± 30.1 [38.8–64.6]	49.4 ± 31.5 [40.3–58.5]	50.3 ± 30.3 [43.1–57.5]	0.815
KOOS—Quality of life	Preoperative	19.5 ± 21.4 [10.3–28.7]	23.4 ± 22.3 [16.9–29.9]	22.0 ± 21.5 [16.9–27.1]	0.562
	Postoperative	50.1 ± 35.7 [34.8–65.4]	49.9 ± 33.1 [40.3–59.5]	50.0 ± 33.2 [42.1–57.9]	0.990
Lysholm score	Preoperative	39.8 ± 24.3 [29.4–50.2]	48.3 ± 22.1 [41.9–54.7]	45.3 ± 22.7 [39.9–50.7]	0.143
	Postoperative	78.3 ± 21.6 [69.0–87.6]	72.7 ± 26.2 [65.1–80.3]	74.8 ± 24.2 [69.0–80.6]	0.481
SKV score	Preoperative	39.7 ± 20.2 [31.0–48.4]	30.0 ± 21.1 [23.9–36.1]	33.6 ± 20.8 [28.7–38.5]	0.101
	Postoperative	78.2 ± 22.0 [68.8–87.6]	70.6 ± 21.3 [64.4–76.8]	73.5 ± 20.7 [68.6–78.4]	0.155
UCLA activity Score	Preoperative	6.1 ± 2.3 [5.1–7.1]	5.5 ± 2.6 [4.8–6.2]	5.7 ± 2.5 [5.1–6.3]	0.417
	Postoperative	6.9 ± 1.9 [6.1–7.7]	6.9 ± 2.7 [6.1–7.7]	6.9 ± 2.4 [6.3–7.5]	0.584
NRS pain (0–10)	Preoperative	6.1 ± 2.7 [5.0–7.2]	6.4 ± 2.0 [5.8–7.0]	6.3 ± 2.2 [5.8–6.8]	0.661
	Postoperative	2.5 ± 2.5 [1.4–3.6]	2.9 ± 2.3 [2.2–3.6]	2.8 ± 2.3 [2.3–3.3]	0.529

*Note*: Data presented as mean ± SD [95% confidence interval].

Abbreviations: ADL, activities of daily living; KOOS, Knee injury and Osteoarthritis Outcome Score; NRS, numerical rating scale; SD, standard deviation; SKV, simple knee value; UCLA, University of California Los Angeles.

No cases of radiographic correction loss, implant failure, delayed union or revision surgery were observed during follow‐up in either group.

## DISCUSSION

The principal finding of this study was that distal femoral osteotomies complicated by hinge fracture and stabilised using percutaneous compression screw fixation demonstrated radiographic and clinical outcomes comparable to osteotomies without hinge fracture. The magnitude of coronal correction, maintenance of alignment at final follow‐up, radiographic union rates and patient‐reported outcome measures were similar between groups. These results indicated that hinge fracture, when stabilised appropriately, did not compromise osteotomy correction or healing.

Hinge integrity is known to play a critical biomechanical role during DFO, serving as the rotational fulcrum during angular correction and contributing to construct stability. Disruption of the hinge may theoretically alter the mechanical environment at the osteotomy site, potentially leading to instability, loss of correction or impaired healing [20, 27, 29, 32]. The addition of a biplanar osteotomy cut further contributed to construct stability by increasing bone contact surface area and improving rotational resistance, which may partially compensate for hinge disruption. Previous studies have emphasised the importance of hinge preservation; Rupp et al. demonstrated that hinge disruption was associated with increased risk of nonunion and correction loss in lateral closing‐wedge DFO [27], and Fujita et al. highlighted its potential impact on mechanical stability and correction accuracy [6]. Despite these concerns, the present study demonstrated maintenance of correction and complete radiographic union in all cases, including those with hinge fracture treated with compression screw fixation, suggesting that supplemental fixation may effectively compensate for cortical disruption [8].

Significant improvements were observed in KOOS, Lysholm score and SKV in both groups, with no differences at final follow‐up. Improvements exceeded the established MCID thresholds for all outcome measures in both groups. These findings were consistent with previous studies demonstrating favourable functional outcomes following DFO when alignment correction was achieved and maintained [14, 34] and suggested that hinge fracture, when appropriately stabilised, did not adversely affect functional recovery.

Hinge fractures occurred in 31% of cases in the present series, consistent with previously reported incidence rates of 10% to 57% [6, 31]. Several risk factors have been identified, including hinge location, bone quality and magnitude of correction [12, 16, 21]. Compression screw fixation represented a simple and reproducible stabilisation technique performed intraoperatively to restore hinge continuity [7]. All hinge fractures were identified and stabilised intraoperatively; no secondary fixation procedures were required. In the present series, a distal ascending trajectory was preferred over a proximal approach, as the latter risks intraarticular penetration and conflicts with plate positioning, while the ascending trajectory provided adequate cortical stabilisation as previously demonstrated in high tibial osteotomy models [8].

Toe‐touch weight‐bearing was maintained for the first 3 weeks postoperatively in the hinge fracture group, followed by progressive partial weight‐bearing until 6 weeks, after which full weight‐bearing was permitted. This protocol was not associated with adverse radiographic or clinical outcomes. Previous studies have recommended delayed weight‐bearing—generally defined as non‐weight‐bearing or toe‐touch weight‐bearing beyond 6 weeks—in cases of hinge fracture due to concerns regarding mechanical instability [3, 20]. The present results suggested that restoration of hinge stability through compression screw fixation may allow earlier and safe progression of weight‐bearing compared to these more conservative protocols.

Several limitations should be acknowledged. The retrospective design may have introduced selection bias, and the hinge fracture group was relatively small. Interobserver reliability was not formally assessed, representing a methodological limitation; however, intraobserver reliability was excellent, as reported in the Methods section. Osteoarthritis severity differed significantly between groups at baseline, which may have influenced functional outcomes despite comparable patient reported outcome measures improvements. This study was conducted at a single institution, which may limit generalisability. Longer‐term follow‐up would be valuable to assess durability of correction and clinical outcomes over time.

## CONCLUSION

DFO‐associated hinge fractures treated with percutaneous compression screw fixation were not associated with inferior radiographic or clinical outcomes at mid‐term follow‐up, supporting this technique as a reliable intraoperative management strategy. In the hinge fracture group, progressive weight‐bearing initiated at 3 weeks postoperatively was not associated with loss of correction or delayed union.

## AUTHOR CONTRIBUTIONS

All authors contributed equally to the conception, design, data collection, analysis, drafting and critical revision of this manuscript. All authors approved the final submitted version.

## CONFLICTS OF INTEREST STATEMENT

Matthieu Ollivier is a paid consultant and receives royalties from Newclip. Matthieu Ollivier is also a paid consultant and receives royalties from Stryker. The remaining authors declare no conflict of interest.

## FUNDING INFORMATION

The authors have no funding to report.

## ETHICS STATEMENT

Ethics approval was obtained from the Institute of Movement and Musculoskeletal System, Department of Orthopaedic Surgery of Sainte‐Marguerite Hospital, France (reference number: CSE_PADS23_021_DGR). Informed consent was obtained from all patients involved in this study.

## Supporting information

Supporting File 1.

## Data Availability

All data used in this study are available from the corresponding author upon reasonable request.
