# Supplementary material for: Percutaneous compression screw fixation for hinge fractures in distal femoral osteotomy does not compromise radiographic or clinical outcomes: A retrospective case‐control study
Source: J Exp Orthop. 2026 Jun 30;13(3):e70830. doi: 10.1002/jeo2.70830 (PMC13317789; doi:10.1002/jeo2.70830)
Supplement: Supplementary file 1 — Supporting File 1. [file JEO2-13-e70830-s001.docx]

**Appendix 1. Surgical Technique: Distal Femoral Osteotomy and Management of Hinge Fracture**

**Lateral Opening-Wedge or Closing-Wedge Distal Femoral Osteotomy**

A longitudinal lateral approach to the distal femur is performed, cantered over the lateral femoral condyle and extending proximally along the distal femoral diaphysis. Following skin incision, blunt dissection is carried down to the iliotibial band, which is incised longitudinally in line with its fibres. The vastus lateralis muscle is elevated subperiosteally and retracted anteriorly using a curved Hohmann or Merle d’Aubigné retractor, allowing exposure of the lateral distal femoral metaphysis. Care is taken to preserve soft tissue attachments posteriorly. A radiolucent Hohmann retractor is then positioned at the posterior aspect of the femoral metaphysis at the level of the planned osteotomy to protect the posterior neurovascular structures and facilitate fluoroscopic visualization.

When performing a freehand osteotomy, two Kirschner wires (1.5–2.0 mm diameter) are inserted from lateral to medial under fluoroscopic guidance. These wires are oriented toward the adductor tubercle, converging approximately 15–20° in the axial plane and positioned at the same vertical level, as confirmed on anteroposterior fluoroscopic imaging. These wires define the primary osteotomy plane and guide subsequent saw cuts. In closing-wedge osteotomy, two additional parallel Kirschner wires are inserted proximally, at a distance determined by preoperative planning, to define the height of the bone wedge to be resected. These proximal wires are oriented toward the same hinge point to ensure accurate angular correction.

When patient-specific instrumentation (PSI) is used, the cutting guide is positioned on the distal femur according to the preoperative three-dimensional plan, ensuring accurate positioning relative to anatomical landmarks. In most cases, a hinge protection pin is inserted at the planned medial hinge point to reduce stress concentration during angular correction and minimize the risk of hinge fracture.

The osteotomy is performed using an oscillating saw under continuous fluoroscopic control. The saw cut is advanced carefully toward the medial cortex, preserving an intact medial cortical hinge measuring approximately 5 to 10 mm in thickness. A biplanar osteotomy is performed by adding an ascending anterior cut in the frontal plane, which improves rotational stability and increases bone contact surface area. In closing-wedge osteotomy, the planned bone wedge is removed, and gradual osteotomy closure is achieved by applying gentle axial pressure through the limb with the knee in full extension, allowing controlled deformation at the hinge site.

In opening-wedge osteotomy, gradual distraction is performed using calibrated osteotomy wedges or spreaders inserted into the osteotomy site. Distraction is increased incrementally until the desired correction is achieved, as confirmed by fluoroscopy. Once the planned opening is obtained, structural bone graft or allograft wedge is inserted to maintain the correction and provide structural support. The definitive fixation plate is then applied and secured using locking screws, ensuring stable fixation. Fluoroscopic imaging is used throughout the procedure to confirm osteotomy orientation, hinge integrity, and final alignment.

Hinge fractures most commonly occur during the saw cut or during angular correction when excessive stress is applied to the medial cortex. Careful control of osteotomy depth and gradual correction reduce this risk.

**Medial Opening-Wedge or Closing-Wedge Distal Femoral Osteotomy**

A medial subvastus minimally invasive approach to the distal femur is performed, cantered over the medial femoral condyle and extending proximally along the distal femoral shaft. A longitudinal skin incision of approximately 8 to 10 cm is made starting at the medial epicondyle and extending proximally. Subcutaneous dissection is performed to expose the fascia of the vastus medialis. The fascia is incised longitudinally in line with muscle fibres, and the vastus medialis muscle is elevated and retracted anteriorly using a curved Hohmann retractor. Posterior soft tissue structures, including the adductor magnus tendon, are preserved.

Careful dissection is performed at the posterior aspect of the femur, and a radiolucent Hohmann retractor is positioned against the posterior cortex at the level of the planned osteotomy to protect the neurovascular bundle, including the superficial femoral artery. The osteotomy plane is defined using Kirschner wires inserted under fluoroscopic guidance toward the lateral hinge point, located at the metaphyseal–epiphyseal junction near the lateral femoral epicondyle. A protective hinge pin may be inserted to reduce stress concentration during correction.

The osteotomy is performed using an oscillating saw under fluoroscopic guidance, preserving a lateral cortical hinge measuring approximately 5 to 10 mm. Biplanar osteotomy is performed as described for the lateral approach to improve rotational stability and facilitate bone healing. Correction is achieved either by wedge removal in closing-wedge osteotomy or by gradual distraction in opening-wedge osteotomy. Definitive fixation is achieved using anatomically contoured locking plates.

**Management of Hinge Fracture**

When hinge fracture is identified intraoperatively, stabilization is performed immediately to restore mechanical continuity and maintain osteotomy stability. Under fluoroscopic guidance, a guide wire is inserted percutaneously across the hinge fracture site, ensuring optimal positioning across the disrupted cortical region. A headless compression screw, typically 4.0 mm in diameter and 40 to 60 mm in length depending on femoral size, is then inserted over the guide wire to achieve compression and stabilize the fracture. Screw placement is confirmed fluoroscopically in multiple planes to ensure appropriate positioning and avoidance of intra-articular penetration.

Compression screw fixation restores cortical stability at the hinge, allowing secure osteotomy fixation and facilitating bone healing.
